# Au/ZnO/In_2_O_3_ nanoparticles for enhanced isopropanol gas sensing performance†

**DOI:** 10.1039/d3ra07507a

**Published:** 2024-01-18

**Authors:** Yuhong Zhang, Lvqing Wang, Shenghui Li, Shengjue Yang, Hang Liu

**Affiliations:** a School of Electrical and Computer Engineering, Jilin Jianzhu University Changchun 130118 China zhangyuhong@jlju.edu.cn

## Abstract

In this paper, a series of Au/ZnO/In_2_O_3_ nanoparticles are synthesized by a facile one-step hydrothermal method. The gas sensing properties of Au/ZnO/In_2_O_3_ materials are investigated in detail. The response of 2%Au/1%ZnO/In_2_O_3_ material to isopropanol increases to six times that of pure In_2_O_3_ materials. In contrast to a pure In_2_O_3_ sensor, the optimal working temperature of the 2%Au/1%ZnO/In_2_O_3_ sensor decreases to 40 °C. The sensing mechanism of Au/ZnO/In_2_O_3_ nanoparticles is mainly explained through the influence of the n–n heterojunction formed by In_2_O_3_ and ZnO. In addition, the introduction of Au contributes to an increase in the gas response. A possible reason is that the introduction of Au produces smaller sized particles on the sensor surface, creating a larger surface area, enhancing the response.

## Abstract 2

In this paper, a series of Au/ZnO/In_2_O_3_ nanoparticles are synthesized by a facile one-step hydrothermal method.

## Graphical abstract

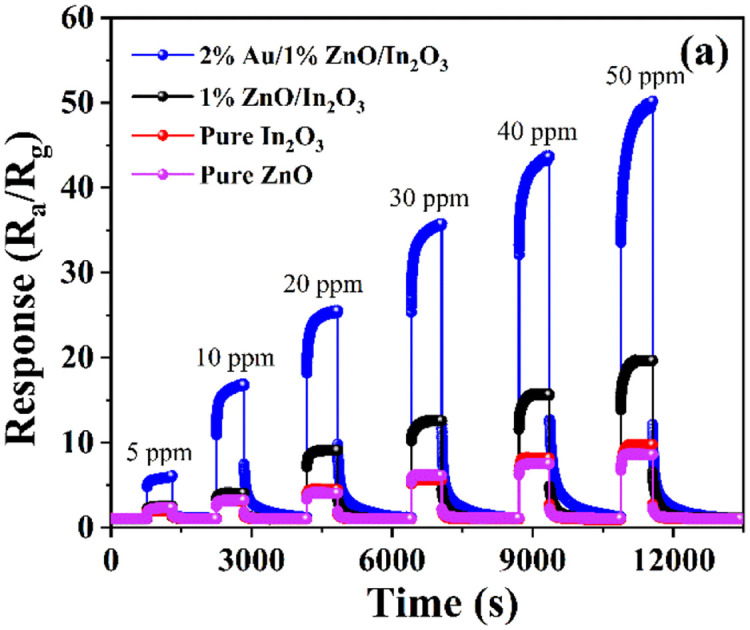

## 1 Introduction

Recently, many industrial exhaust gases have been generated with the development of industry, which will continuously contaminate the air and harm human health.^1^ Isopropanol (C_3_H_8_O), a typical volatile organic compound (VOC) gas, has attracted attention and has been used in many fields, such as pharmaceuticals, cosmetics, plastics, spices, and coatings.^2,3^ In particular, isopropanol is slightly toxic and shows a certain carcinogenicity, so the World Health Organization (WHO) lists it as a category-III carcinogen.^3,4^ When the concentration of isopropanol is less than 400 ppm, some symptoms such as dizziness and vomiting can be observed and can even irritate the eyes and respiratory tract, causing discomfort, while internal bleeding, high blood pressure and paralysis of the central nervous system will occur at high levels that can cause grave danger to humans.^5,6^ To sum up, the effective detection of isopropanol is undoubtedly crucial.

In the last few years, metal oxide semiconductor (MOS) gas sensors have shown plenty of attractive advantages compared to other gas detection equipment that is excessively bulky, which is making people pay more and more attention to this type of sensor.^3^ Therefore, MOS sensors with advantages, such as low working temperature, high sensitivity, simple operation, and low cost have gradually become the guiding ideology for future exploration into sensors.^7–9^ So far, people have developed a lot of MOS sensors based on ZnO,^10^ In_2_O_3_,^11^ SnO_2_,^12^ and NiO.^13^ However, in terms of sensitivity, response time and other aspects, MOS gas sensors are still not suitable to satisfy market demand; that is, they need to be improved and explored through other methods.^14,15^ Liu *et al.* have prepared an In_2_O_3_/ZnO composite with a fern-like mesoporous structure *via* a simple template method and measured the sensing characteristics, which showed that the optimal response is 44.6 (*S* = *R*_a_/*R*_g_) for triethylamine at 100 ppm.^16^ Hou *et al.* reported that a Cd-doped In_2_O_3_ gas sensor produced by a facile solvothermal method displayed a sensitivity of 20.12 (*S* = *R*_a_/*R*_g_) under 100 ppm acetone at the optimized operating temperature.^17^ Bai *et al.* also reported an isopropanol gas sensor based on 4 at% Ce/In_2_O_3_ nanosheets, which showed a gas sensitivity of 93 (*S* = *R*_a_/*R*_g_) towards 100 ppm isopropanol at an optimized working temperature of 220 °C.^18^ As mentioned above, even though many scientific research teams are researching sensors based on In_2_O_3_, there are still constraints, such as high operating temperature and low sensitivity. It is well known that In_2_O_3_ is a typical n-type semiconductor that exhibits good electrical conductivity and high photochemical stability, with a wide bandgap (3.5–3.7 eV), and it is a candidate for a gas sensor.^19–21^ Due to these advantages, In_2_O_3_ is widely used in the field of gas sensors. Nevertheless, it is necessary to improve the gas sensing characteristics of pure In_2_O_3_ by controlling its morphology or changing the structure of the material itself.^22–24^ In addition, an n–n heterojunction based on ZnO–In_2_O_3_ may be a useful route for enhancing the response of In_2_O_3_.^25,26^

In this work, a ZnO–In_2_O_3_ n–n heterojunction is fabricated by a simplified one-step hydrothermal method, then it is modified by employing a noble metal, Au. These nanomaterials are used to fabricate isopropanol sensors and their performance is investigated, including sensitivity, optimal temperature, response/recovery time, and selectivity. At the end of the paper, the gas sensing mechanisms that lead to improved performance of isopropanol gas sensors are documented.

## 2 Experimental

### 2.1 Preparation of Au/ZnO/In_2_O_3_ nanoparticles

Pure In_2_O_3_, pure ZnO, *x*mol%ZnO/In_2_O_3_ (*x* = 1,2,3) and *y*mol%Au/1mol%ZnO/In_2_O_3_ (*y* = 1,2,3) are synthesized by a relatively simple hydrothermal method. Raw materials of analytical grade, consisting of indium nitrate hydrate (In(NO_3_)_3_·4.5H_2_O, 99.9%), zinc nitrate hexahydrate (Zn(NO_3_)_2_·6H_2_O, 99%), chloroauric acid (HAuCl_4_·4H_2_O, ≥47.8% (the content of Au)), and anhydrous ethanol (C_2_H_6_O, 99.7%), were supplied by Sinopharm Chemical Reagent Co. Ammonia (NH_3_, 25–28w/%) was supplied by the Tianjin Guangfu Technology Development Co. The synthetic path of pure In_2_O_3_ and ZnO/In_2_O_3_ was as follows. First, 0.1 M In(NO_3_)_3_·4.5H_2_O was added to 15 mL of deionized water, and Zn(NO_3_)_2_·6H_2_O (0 mM, 0.05 mM, 0.1 mM, 0.3 mM) was added to 15 mL of deionized water. After 20 minutes of separate stirring, the two solutions were mixed with the addition of ammonia. Then, the mixture was put into an Teflon-lined stainless-steel autoclave. It was kept in a desiccator for 20 h at 180 °C. After cooling, centrifugation of the reaction solution was carried out. It was washed alternately with deionized water and ethanol. Then, the sample was dried at 80 °C for 10 h in a drying oven. Finally, the sample was calcined in a muffle furnace to obtain pure In_2_O_3_ and *x*mol%ZnO/In_2_O_3_ (*x* = 0.5,1,3) at 500 °C.

Nanoparticles of *y*mol%Au/1mol%ZnO/In_2_O_3_ were obtained by modifying 1 mol%ZnO/In_2_O_3_ with different molar ratios of HAuCl_4_·4H_2_O. Three portions of 0.572 g of In(NO_3_)_3_·4.5H_2_O and 0.004 g of Zn(NO_3_)_2_·6H_2_O were weighed and added to a beaker containing 15 mL of deionized water. Different amounts of Au (0.006, 0.012 and 0.018 g) were weighed and added to the three beakers and stirred continuously during the process. The rest of the steps are consistent with the preparation of ZnO/In_2_O_3_; that is, *y*mol%Au/1mol%ZnO/In_2_O_3_ (*y* = 1, 2, 3) samples were synthesized.

### 2.2 Characterization

The crystal phase of the fabricated materials was analyzed by X-ray diffraction (XRD, Rigaku Ultima IV X-ray Diffractometer) with Cu Kα1 radiation (*λ* = 0.154056 nm, 40 kV, 100 mA). The morphology of the materials was scanned using scanning electron microscopy (SEM, FEI QUANTA FEG 450). The elemental composition was analyzed by energy dispersive spectroscopy (EDS, OXFORD Xplore). An inductively coupled plasma optical emission spectrometer r(ICP-OES, Agilent 725) was used to evaluate the concentration of Au and Zn in In_2_O_3_ samples. Gas-sensitive properties were tested with a Chemical Gas Sensor-8 Intelligent Gas Sensing Analysis System (CGS-8, SINO AGGTECH).

The isopropanol gas sensor consisted of a prepared sample, a ceramic tube with two gold electrodes and four Pt wires, a heating wire of Ni–Cr alloy used to manage the operating temperature, and a hexagonal base. First, a small amount of material was put into a grinding bowl, then a small amount of deionized water was added, with anhydrous ethanol to make it into a paste. Finally, the paste sample was coated onto the ceramic tube. Then. the sensor was annealed in a muffle furnace at 400 °C. The final step was to weld the ceramic tube with a gas-sensitive layer on the base, and it was inserted into an aging table for aging until the resistance stabilized. After everything was complete, various performance studies of the sensor could be carried out.

In this paper, the target gas used is initially a volatile liquid, and the final measured gas is obtained after liquid gas distribution. The formula is shown as eqn (1):1
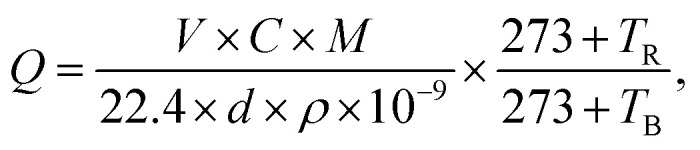
where *Q* is the volume of the injected liquid (ml), *V* is the volume of the test chamber (ml), *M* is the molecular weight of the substance (g), *d* is the purity of the liquid, *C* is the concentration of the gas to be prepared (ppm), *ρ* is the density of the liquid (g cm^−3^), and *T*_R_ and *T*_B_ are the testing temperature and room temperature, respectively. In addition, the process of gas testing is shown in Fig. S1.† First, the corresponding concentration of gas is pumped into a glass bottle using a syringe. Then, the sensor is quickly put into the glass bottle and the response is observed.

## 3 Results and discussion

### 3.1 Characterization

**Fig. 1:**
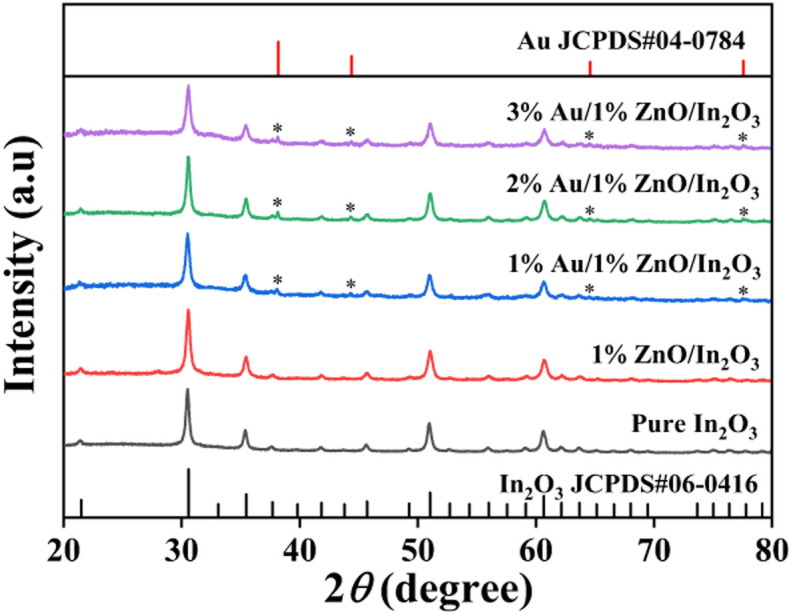
XRD spectra of In_2_O_3_, 1%ZnO/In_2_O_3_ and *x*%Au/1%ZnO/In_2_O_3_ (*x* = 1, 2, 3) samples.

Herein, the XRD schemes of all fabricated samples are displayed in Fig. 1. The peaks of strong diffraction for each of the five samples are in perfect agreement with the standard card (JCPDS # 06-0416) for pure ln_2_O_3_, corresponding to (211), (222), (400), (411), (332), (431), (440), (611), and (622) planes at 21.501°, 30.524°, 35.481°, 37.577°, 41.835°, 45.647°, 51.112°, 56.005° and 60.643°, respectively. The XRD patterns of the 1%ZnO/In_2_O_3_ sample are similar to the standard card for pure In_2_O_3_. The diffraction peaks of ZnO are dismissed due to the low doping amount of ZnO. However, for Au/ZnO/In_2_O_3_ samples, the diffraction peaks of Au are obtained, which correspond to (111), (200), (220), and (311) planes at 38.212°, 44.416°, 64.601° and 77.55°, respectively. There are no other excess peaks, indicating that all prepared samples are pure phase. The XRD schemes of pure ZnO and *x*%ZnO/In_2_O_3_ (*x* = 0.5, 1, 3) are matched with the corresponding standard cards, as shown in Fig. S2.†

**Fig. 2:**
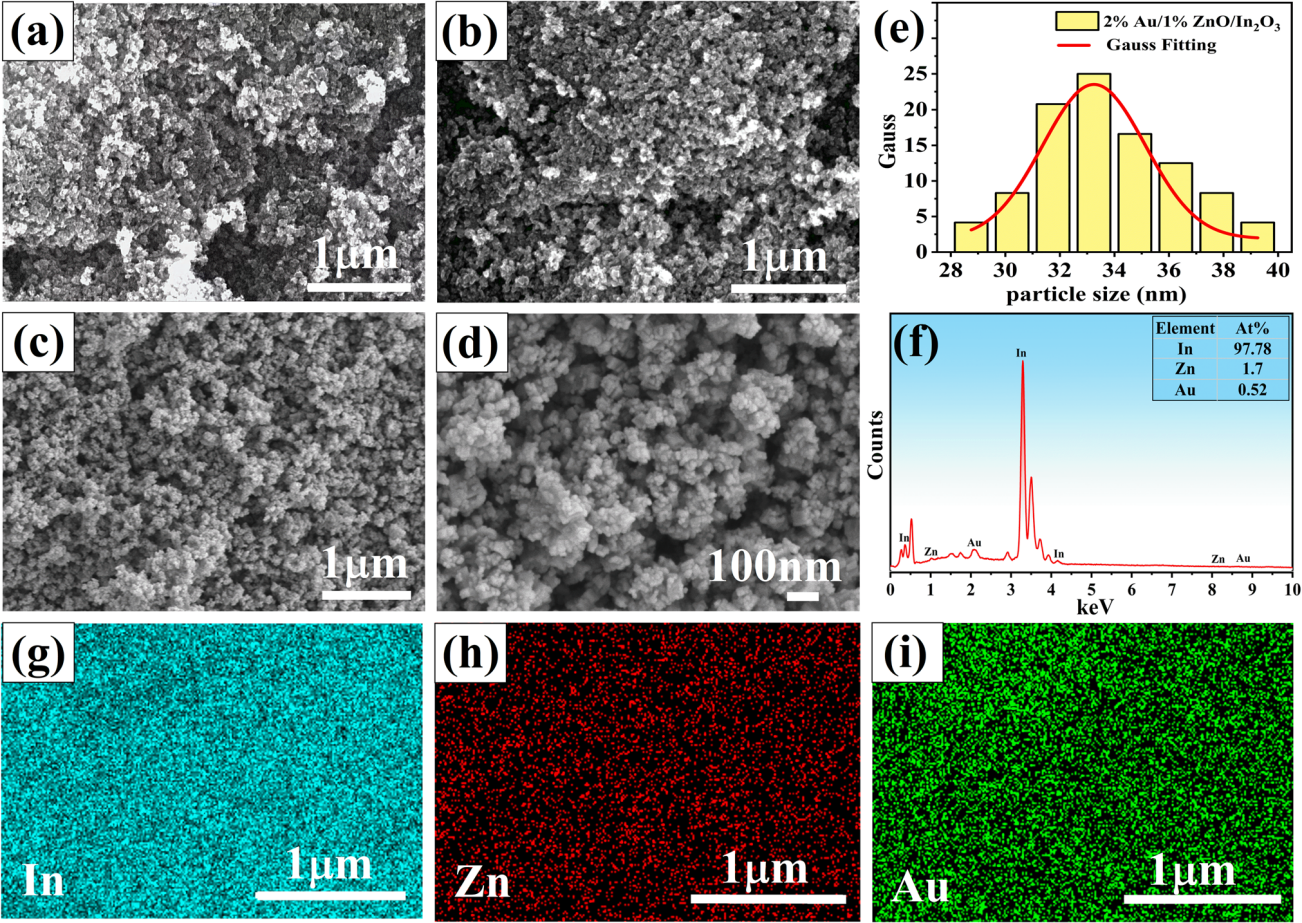
SEM of (a) pure In_2_O_3_, (b) 1%ZnO/In_2_O_3_, (c) and (d) 2%Au/1%ZnO/In_2_O_3_; (e) size distribution of 2%Au/1%ZnO/In_2_O_3_ nanoparticles; (f)–(i) EDS of the 2%Au/1%ZnO/In_2_O_3_ sample.

The SEM of In_2_O_3_, 1%ZnO/In_2_O_3_ and 2%Au/1%ZnO/In_2_O_3_ nanoparticles at the 1 μm scale are displayed in Fig. 2(a)–(c). It can be seen that the morphology of pure In_2_O_3_ presents agglomerating nanoparticles in Fig. 2(a) and that of the 1%ZnO/In_2_O_3_ sample becomes looser in Fig. 2(b). Once Au is doped, the SEM image of 2%Au/1%ZnO/In_2_O_3_ becomes loose and porous in Fig. 2(c). Fig. 2(d) shows 2%Au/1%ZnO/In_2_O_3_ nanoparticles with a size of 100 nm, and the loose and porous structure of the sample can be clearly seen. A histogram is used to present the distribution of particle sizes, with a Gaussian fit to a red curve in Fig. 2(e). This confirms an average particle size of 33.23 nm for 2%Au/1%ZnO/In_2_O_3_ nanoparticles. In addition, the morphology of the Au/ZnO/In_2_O_3_ sample is adjusted through the catalysis of Au and ZnO.

The elemental distributions of the 2%Au/1%ZnO/In_2_O_3_ sample are tested in Fig. 2(f)–(i). It can be clearly seen in In, Au and Zn. The result indicates that ZnO and noble metal Au have been successfully loaded in In_2_O_3_. Fig. 2(f) shows the EDS semi-quantitative analytical spectrum, which determines the atomic percentage of the elements contained in the final product, *i.e.*, 97.78% for In, 1.7% for Zn, and 0.52% for Au, which also proves the successful preparation of Au/ZnO/In_2_O_3_.

The doping concentration of Au and Zn was analyzed by ICP-OES, and the results are shown in Table S1.† It is obvious that the observed concentrations of the Au are 0.65 mol%, 1.46 mol% and 2.45 mol%, and the concentrations of Zn are 0.73 mol%, 0.76 mol% and 0.72 mol%. Thus, the results are suitable for further studies.

### 3.2 Gas sensing performance of Au/ZnO/In_2_O_3_ nanoparticles

**Fig. 3:**
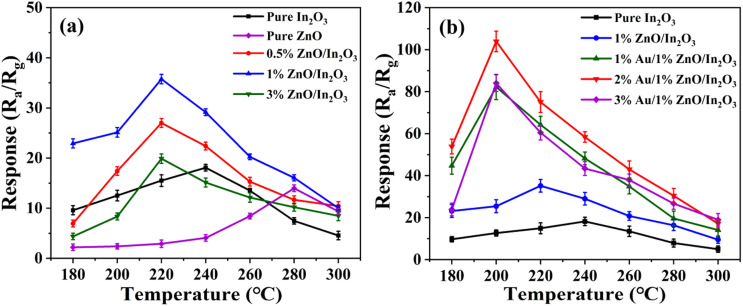
Comparison graph of the response and optimal working temperature of (a) pure In_2_O_3_, pure ZnO and *x*%ZnO/In_2_O_3_ (*x* = 1, 2, 3) and (b) pure In_2_O_3_, 1%ZnO/In_2_O_3_ and *y*%Au/1%ZnO/In_2_O_3_ (*y* = 1, 2, 3) for 100 ppm isopropanol.

The gas sensing properties of pure In_2_O_3_, pure ZnO, ZnO/In_2_O_3_ and Au/ZnO/In_2_O_3_ for isopropanol are illustrated in detail. The responses of pure In_2_O_3_, pure ZnO and *x*%ZnO/In_2_O_3_ (*x* = 1, 2, 3) to 100 ppm of isopropanol were measured at 180–300 °C, and the optimum operating temperature for the gas sensor was determined, as presented in Fig. 3(a). The sensitivities of pure In_2_O_3_ and pure ZnO sensors reach their maximum values (18 and 14) at 240 °C and 280 °C, respectively. Surprisingly, the response of the ZnO/In_2_O_3_ sample is enhanced, and its optimal working temperature is reduced. The response of the 1%ZnO/In_2_O_3_ sensor reached 35.5 at an optimal working temperature of 220 °C, which is twice as high as that of pure In_2_O_3_. The ZnO/In_2_O_3_ composite has an n–n heterojunction structure. According to a previous report, the construction of an n–n heterojunction helps to boost the responsiveness of gas sensors. To explore the sensing performance of ZnO/In_2_O_3_ samples, the Au/ZnO/In_2_O_3_ sample was designed, and the responses of *y*%Au/1%ZnO/In_2_O_3_ (*y* = 1, 2, 3) were tested, as shown in Fig. 3(b). The sensitivity of the 2%Au/1%ZnO/In_2_O_3_ isopropanol gas sensor reached 110, an improvement of more than 6 times that of pure In_2_O_3_.

The reasons for the increase in sensitivity and decrease in optimum operating temperature of Au/ZnO/In_2_O_3_ sample are discussed as follows. For the optimum operating temperature, the temperature curve of all samples is volcanic, as shown in Fig. 3. Inadequate chemical activation at lower temperature leads to an inert reaction, which prevents them from adsorbing onto the surface of the gas-sensitive material, whereas, when the temperature is excessive, the gas molecules spill out before reacting with the adsorbed oxygen due to their high activation energy, thus the sensitivity is the highest only at the optimal working temperature.^27–29^ The optimal working temperature of pure In_2_O_3_ decreases continuously with the doping of ZnO and Au, which is the same situation as that observed by Wang *et al.* and Ma *et al.*^30,31^ The reason may be the lowering of the grain boundary potential barrier due to doped Au/ZnO and higher activation energy of the Au/ZnO/In_2_O_3_ sample. For sensitivity, the n–n heterojunction structure of ZnO/In_2_O_3_ can form a wider electron depletion layer, greatly improving the gas sensor response to the target gas;^18^ the response of the Au/ZnO/In_2_O_3_ sample is further increased. The results may be induced by the catalysis of Au. The looser nanoparticles can contribute to more absorbed oxide, which can increase the active sites involved in the reaction.^12^

**Fig. 4:**
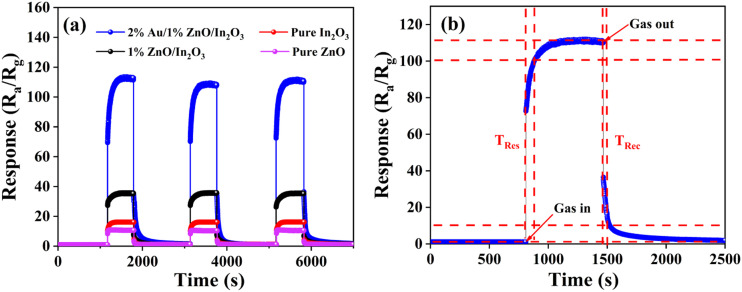
(a) Dynamic response–recovery cycle curves of gas sensors for 100 ppm isopropanol gas at their respective optimal operating temperatures. (b) Response–recovery time curve of the 2%Au/1%ZnO/In_2_O_3_ sensor.

Furthermore, the response–recovery times are key parameters for a gas sensor. The three cycle curves of response–recovery of four sensors for 100 ppm isopropanol were measured and are shown in Fig. 4(a). The outcomes demonstrate the remarkable reproducibility of the four sensors. Generally, response time (*T*_Res_) and recovery time (*T*_Rec_) are the times required for the gas sensor resistance to reach 90% of the total change in the value when the target gas is adsorbed and desorbed, respectively. When the response of the sensor reaches a certain value, there is no longer a significant upward trend; that is to say, the sensor is judged to be fully responsive to the target gas. The response–recovery times of the 2%Au/1%ZnO/In_2_O_3_ sensor are shown in Fig. 4(b), where the response time is 78 seconds and the recovery time is 49 seconds.

**Fig. 5:**
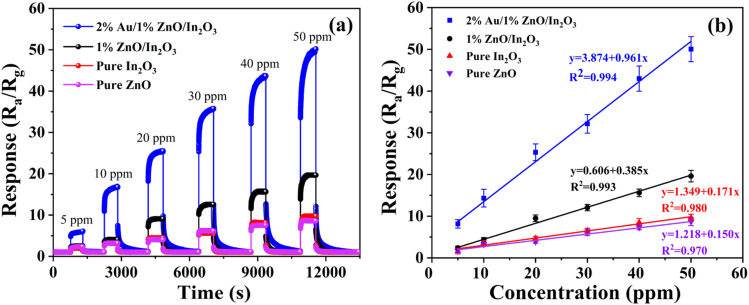
(a) Response of sensors to different concentrations of isopropanol at their respective optimal operating temperatures. (b) Linear fit curves of the response to the concentration of isopropanol.

The dynamic response–recovery cycle curves of the gas sensors for various gas contents (5 ppm, 10 ppm, 20 ppm, 30 ppm, 40 ppm, and 50 ppm) of isopropanol at their corresponding optimal operating temperatures are illustrated in Fig. 5(a). It can be seen that the response values of the 2%Au/1%ZnO/In_2_O_3_ gas sensor have a much higher response than all other sensors. The linear fitting curves between isopropanol concentration and response for the four sensors are shown in Fig. 5(b). The error bar in the figure is the error obtained after repeating the experiment three times. The fitted correlation coefficients *R*^2^ for 2%Au/1%ZnO/In_2_O_3_, 1%ZnO/In_2_O_3_, pure In_2_O_3_ and pure ZnO are 0.994, 0.993, 0.980 and 0.970, respectively. Their values are very close to 1, which means the fit of the sensor conforms to the linear law.

**Fig. 6:**
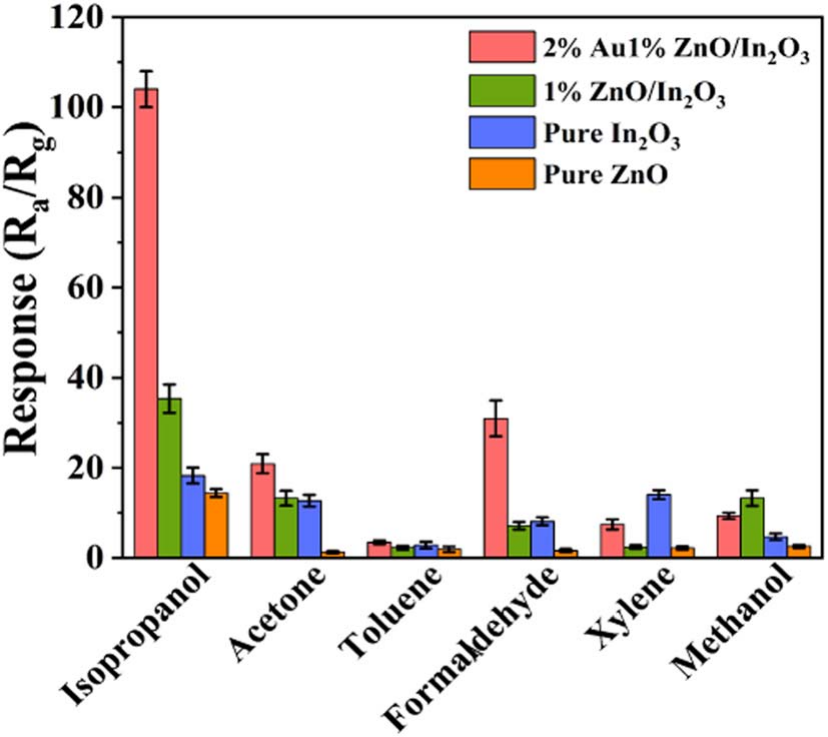
Selectivity of sensors to 100 ppm isopropanol, acetone, toluene, formaldehyde, xylene, and methanol.

The selectivity of the gas sensor for different gases is also a particularly important aspect. The sensitivities of pure In_2_O_3_, pure ZnO, 1%ZnO/In_2_O_3_ and 2%Au/1%ZnO/In_2_O_3_ sensors for different VOC gases (isopropanol, acetone, toluene, formaldehyde, xylene, and methanol) were tested and are shown in Fig. 6. The concentration of all test gases is 100 ppm. Compared with all tested VOC gases, the Au/ZnO/In_2_O_3_ sensors show a significant advantage in response to isopropanol. Subsequently, the order of 2%Au/1%ZnO/In_2_O_3_ response from strong to weak is isopropanol (104.5) > formaldehyde (31.1) > acetone (22.3) > methanol (9.8) > xylene (8.5) > toluene (2.2). The response of 2%Au/1%ZnO/In_2_O_3_ to isopropanol is 47.5 times higher than that to toluene, demonstrating excellent gas selectivity. The higher selectivity to isopropanol might be associated with the bond dissociation energy and amounts of electrons released for a one-molecule reaction. The lower C–C bonding energy of isopropanol than those of the O–H bond and C–O bond enhances the oxygen reaction and dehydration of isopropanol. Moreover, a single isopropanol molecule can expend more adsorbed O^−^ and release more electrons (e^−^) back to the conduction band, which induces a lot of change in resistance. The above effect will lead to a high response to isopropanol.

Finally, the prepared gas sensor is compared with other sensors in Table 1. It is found that other gas sensors either have higher optimum operating temperatures or lower sensitivity, but the isopropanol sensor based on pure In_2_O_3_ doped with pure ZnO and modified with noble metal Au in this study has the merits of superior sensitivity and lower optimal operating temperature.

**Table 1:** Comparison of gas sensing properties of various materials for target gases

Materials	Temp. (°C)	Target gases	Conc.(ppm)	Response (*R*_a_/*R*_g_)	Ref.
Ce–In_2_O_3_	220	Isopropanol	100	93	18
NiO/NiCo_0.06_Fe_1.94_O_4_	183.5	Isopropanol	100	11.2	32
BCO/In(OH)_3_·*x*H_2_O	100	Isopropanol	100	20.39	33
In_2_O_3_	260	Hydrogen	500	18	34
ZnO/In_2_O_3_	240	Xylene	50	16	35
Fe–In_2_O_3_	350	Ethanol	100	133	36
ZnO@In_2_O_3_	275	NO_2_	70	68	37
Pure In_2_O_3_	240	Isopropanol	100	18	This work
Au/ZnO/In_2_O_3_	200	Isopropanol	100	110	This work

### 3.3 Gas sensing mechanism

The sensing mechanism of the isopropanol sensor in this work is analyzed as given below. Generally, the most likely mechanism of the MOS gas sensor is based on the change in sensor resistance in air and in the target gas. The equation “*S* = *R*_a_/*R*_g_” represents the sensitivity, where *R*_a_ and *R*_g_ are the resistance of the gas sensor in the atmosphere and in the gas to be measured, respectively. When the gas sensor is in air, oxygen is adsorbed on the surface of the material, oxygen molecules O_2_ (gas) become adsorbed oxygen O_2_ (ads), and the electrons in the conduction band of the material are captured at the optimum operating temperature (*T* < 300 °C) to generate O_2_^−^ and O^−^. When the gas sensor is placed into isopropanol, the gas immediately adsorbs onto the surface of the material and reacts with O_2_^−^ and O^−^ on the surface,^38^ generating H_2_O and CO_2_ and releasing electrons to the surface of the material.

**Fig. 7:**
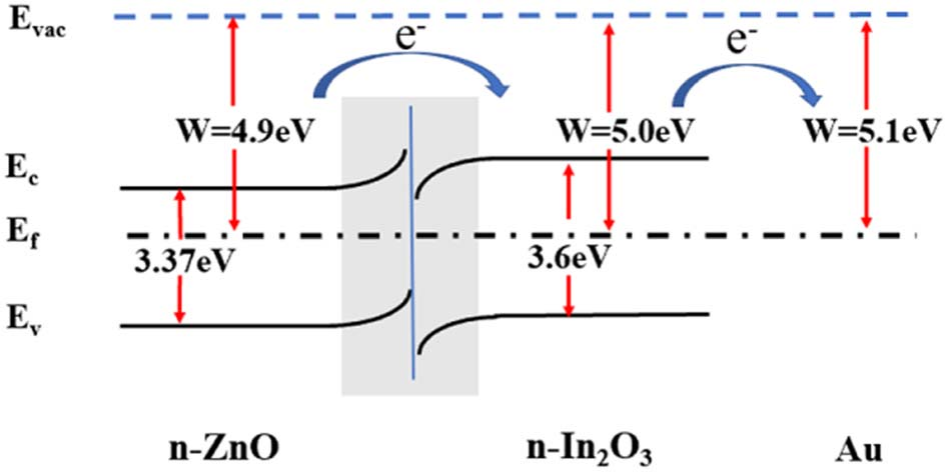
Schematic diagram of the energy band structure of 2%Au/1%ZnO/In_2_O_3_.

The energy level diagram of the Au/ZnO/In_2_O_3_ sensor is shown in Fig. 7. The work function and forbidden bandwidth are 5.0 eV and 3.6 eV for In_2_O_3_ and 4.9 eV and 3.37 eV for ZnO, respectively.^39,40^ Generally, electrons will flow from materials with a low work function (or higher Fermi energy level) to materials with a higher work function (or lower Fermi energy level), and the process continues until the Fermi energy levels of the two substances are in agreement.^41^ In this way, electrons will flow from ZnO to In_2_O_3_, leading to equilibrium of their Fermi energy levels; therefore, the In_2_O_3_ in the heterojunction gains more electrons, making the oxygen easier to adsorb, which helps to promote the properties of the gas sensors.^42^

Finally, the morphology of the sample also has a great influence on the response of the gas sensor. From pure In_2_O_3_ nanoparticles to 2%Au/1%ZnO/In_2_O_3_ nanoparticles, the sample gradually becomes loose and porous, which means that the specific surface area of the sample increases and is more favorable for gas adsorption.

## 4 Conclusion

In conclusion, a series of Au/ZnO/In_2_O_3_ nanoparticles were prepared *via* a hydrothermal method. The XRD patterns and SEM images of the samples have been displayed. The Au/ZnO/In_2_O_3_ sample presents loose and porous nanoparticles. In addition, the gas sensing performances of the Au/ZnO/In_2_O_3_ sensor toward isopropanol were researched. The sensor based on 2%Au/1%ZnO/In_2_O_3_ nanoparticles shows the maximum response to isopropanol. Further, the sensor has a response time of 78 seconds and a recovery time of 49 seconds. The nn heterojunction composed of In_2_O_3_ and ZnO, catalytic effect of Au and loose nanoparticles were used to illustrate the gas sensing mechanism. In sum, the Au/ZnO/In_2_O_3_ nanoparticles are suitable for the design of an isopropanol sensor.

## Conflicts of interest

There are no conflicts to declare.

## Supplementary Material

RA-014-D3RA07507A-s001
