# Supplementary material for: Au/ZnO/In2O3 nanoparticles for enhanced isopropanol gas sensing performance
Source: RSC Adv. 2024 Jan 18;14(5):3044–51. doi: 10.1039/d3ra07507a (PMC10794950; doi:10.1039/d3ra07507a)
Supplement: RA-014-D3RA07507A-s001 [file RA-014-D3RA07507A-s001.pdf]

Electronic Supplementary Material (ESI) for RSC Advances.
This journal is © The Royal Society of Chemistry 2023

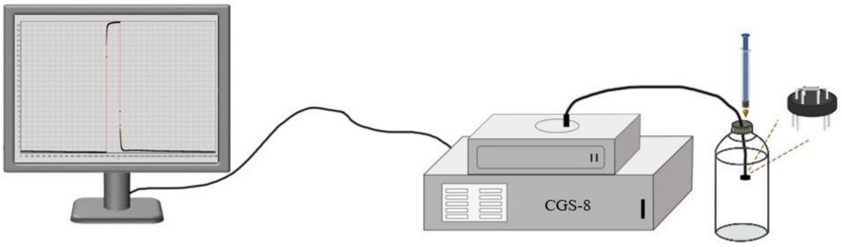

FS1. Schematic of sensor fabrication and gas testing.

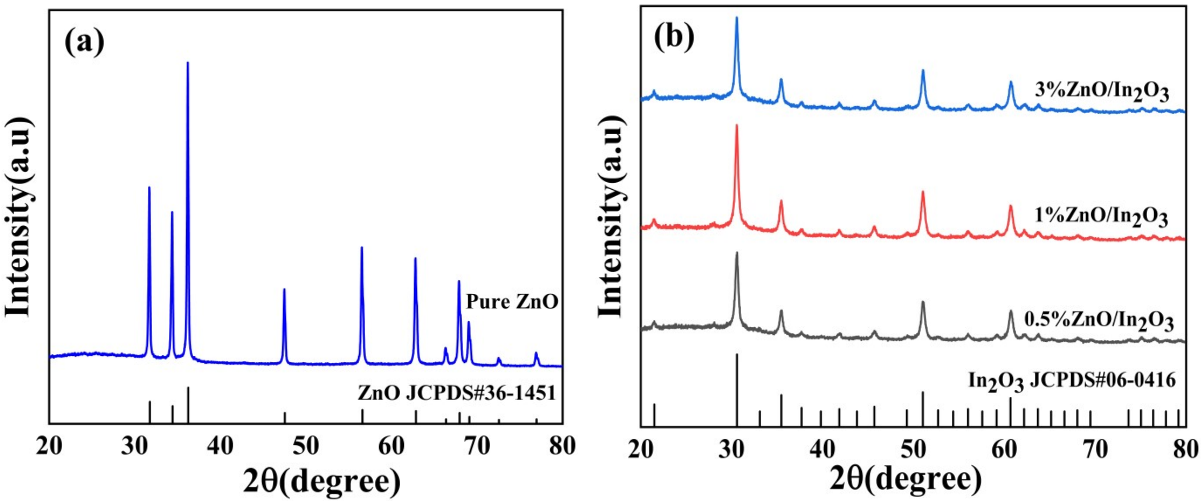

Fig.S2. X-ray diffraction pattern of Pure ZnO, x %ZnO/In_2O_3(x=0.5,1,3) samples.

Table S1 The Au and Zn additional concentrations in In_2O_3 host

sample	1mol %Au/1 mol% ZnO /In_2O_3	2 mol%Au/1 mol% ZnO /In_2O_3	3 mol %Au/1 mol% ZnO /In_2O_3
Au Concentration	0.65	1.46	2.45
Zn Concentration	0.73	0.76	0.72
